# Two new species of the millipede genus *Tylopus* Jeekel, 1968 from Shan State, Myanmar (Diplopoda, Polydesmida, Paradoxosomatidae)

**DOI:** 10.3897/zookeys.1040.66209

**Published:** 2021-05-28

**Authors:** Natdanai Likhitrakarn, Sergei I. Golovatch, Ruttapon Srisonchai, Somsak Panha

**Affiliations:** 1 Division of Plant Protection, Faculty of Agricultural Production, Maejo University, Chiang Mai, 50290, Thailand; 2 Biodiversity and Utilization Research Center of Maejo University, Maejo University, Chiang Mai, 50290, Thailand; 3 Institute for Problems of Ecology and Evolution, Russian Academy of Sciences, Leninsky pr. 33, Moscow 119071, Russia; 4 Department of Biology, Faculty of Science, Khon Kaen University, Khon Kaen, 40002, Thailand; 5 Animal Systematics Research Unit, Department of Biology, Faculty of Science, Chulalongkorn University, Bangkok, 10330, Thailand; 6 Academy of Science, The Royal Society of Thailand, Bangkok 10300, Thailand

**Keywords:** Key, map, Paradoxosomatinae, Sulciferini, taxonomy, *Tylopus monticola* sp. nov., *Tylopus sutchariti* sp. nov.

## Abstract

The predominantly Indochinese to southern Chinese millipede genus *Tylopus* presently comprises 76 described species, including two new, *T. monticola***sp. nov.** and *T. sutchariti***sp. nov.**, both described and illustrated based on material from a limestone mountain in Taunggyi District, Shan State, Myanmar. Both new species have been found to occur syntopically near limestone caves and are assumed to be narrowly endemic to the Taunggyi Mountains, southwestern Shan State, Myanmar. A key to all six *Tylopus* species known to occur in Myanmar is provided, and their distributions are also mapped.

## Introduction

The predominantly Indochinese to southern Chinese millipede genus *Tylopus* Jeekel, 1968, has long been recognized as one of the most speciose and widespread not only within the family Paradoxosomatidae, but also in the entire class Diplopoda (Likhitrakarn et al. 2010, 2016; Golovatch 2019). This genus, formerly known as *Agnesia* Attems, 1953, has been reviewed and rediagnosed several times (Jeekel 1965, 1968; Golovatch and Enghoff 1993; Likhitrakarn et al. 2010), but most taxonomic works have focused on adding new species descriptions, presenting a key and a distribution map to reveal the high diversity of the genus (Nguyen 2012; Liu and Luo 2013, Golovatch 2013, 2014, 2018, 2019, 2020; Likhitrakarn et al. 2014, 2016). At the moment, 74 species of *Tylopus* are known from Indochina and the adjacent parts of southern China and Myanmar (formerly Burma). Most of the known species diversity of *Tylopus* is encountered in Thailand and Vietnam.

Myanmar forms part of the Indo-Burma biodiversity hotspot (Myers et al. 2000; Sodhi et al. 2004). It supports extremely high biodiversity and abundant natural resources, including millipedes (Diplopoda). At present, Myanmar’s known millipede diversity has gradually been revealed to amount to 96 species from 36 genera, 13 families and eight orders, containing 74 endemic and only five widespread synanthropic species (Likhitrakarn et al. 2017, 2018; Pimvichai et al. 2018; Srisonchai et al. 2018a, b). Furthermore, there are 527 millipede records from Burmese amber (Burmite; Cretaceous, ca 100 Mya), representing 13 of the 16 extant orders. Only the orders Sphaerotheriida, Julida and Siphonocryptida have not yet been reported from Burmite. Against this background, no fossil of the family Paradoxosomatidae, one of the largest and most diverse in the entire class Diplopoda globally, has previously been recorded from Myanmar (Wesener and Moritz 2018).

Four *Tylopus* species, all endemic, have been found in Myanmar. The first two species of *Tylopus* to be revealed from that country were *Tylopus doriae* (Pocock, 1895) and *T. silvestris* (Pocock, 1895), both described by Pocock (1895). It was 120+ years later that two further species were added: *T. brehieri* Golovatch, VandenSpiegel & Semenyuk, 2016 and *T. punctus* Likhitrakarn, Golovatch & Panha, 2016 (Golovatch et al. 2016; Likhitrakarn et al. 2016). Myanmar’s climate, geology, topography and, partly, its biota are very similar to those of the neighbouring Thailand; consequently the *Tylopus* species diversity in Myanmar is surprisingly low compared to Thailand with its 31 species. This is undoubtedly due to many areas of Myanmar still being difficult to access, remaining poorly collected and often even dangerous, coupled with local natural history research being rudimentary and secluded. Hardly surprisingly, the arthropod fauna of Myanmar is poorly known and understudied. Studies on the millipede diversity of Myanmar have recently resumed since the British colonial times, chiefly due to the activities of the Animal Systematics Research Unit, Department of Biology, Faculty of Science, Chulalongkorn University, Bangkok, Thailand, headed by one of us (SP).

The present paper puts on record two new species of *Tylopus* collected from a limestone mountain in the Taunggyi District, southwestern Shan State, Myanmar. A key to and updated distributions of all six species of *Tylopus* currently known to occur in Myanmar are also provided.

## Materials and methods

New material was collected in Myanmar, especially in limestone mountain areas, with the support of Fauna & Flora International (**FFI**) in 2015–2017, collaborating with the Animal Systematics Research Unit (**ASRU**), Chulalongkorn University. The collecting activities took place under the limestone conservation projects which aim to protect biodiversity in limestone habitats (Grismer et al. 2018a, b, c; Fauna & Flora International 2021).

Live animals were photographed in the laboratory using a Nikon 700D digital camera with a Nikon AF-S VR 105 mm macro lens. Specimens were preserved in 75% ethanol, and morphological observations were carried out in the laboratory using an Olympus stereo microscope. Scanning electron micrographs (SEM) of gonopods coated with gold were taken using a JEOL, JSM–5410 LV microscope, returned to alcohol after SEM examination. Digital images of the specimens were taken in the laboratory and assembled using the “Cell^D^” automontage software of the Olympus Soft Imaging Solution GmbH package. In addition, line drawings of gonopod characters were also prepared. Both holotypes, as well as most of the paratypes are housed in the Museum of Zoology, Chulalongkorn University (CUMZ), Bangkok, Thailand; some paratypes are donated to the collection of the Zoological Museum, State University of Moscow (ZMUM), Russia, as indicated in the text.

Collecting sites were located by GPS using the WGS84 datum. The distribution maps of all *Tylopus* species recorded from Myanmar were executed using QGIS 3.18.0 (QGIS Development Team 2021). Google satellite maps were downloaded via the QuickMapServices plugin. The images were enhanced and arranged in plates with Adobe Photoshop CS6 software.

In the synonymy sections, D stands for the original description and/or subsequent descriptive notes, K for the appearance in a key, L for the appearance in a species list, and M for a mention.

Terminology concerning gonopodal and somatic structures mostly follows Golovatch and Enghoff (1993) and Likhitrakarn et al. (2010, 2016). Abbreviations of certain gonopodal structures in the figures are explained both in the text and figure captions.

## Taxonomy

### Family Paradoxosomatidae Daday, 1889

**Subfamily Paradoxosomatinae Daday, 1889**

**Tribe Sulciferini Attems, 1898**

#### Genus *Tylopus* Jeekel, 1968

##### Tylopus brehieri

Taxon classificationAnimaliaPolydesmidaParadoxosomatidae

Golovatch, VandenSpiegel & Semenyuk, 2016

C403BD63-D63C-5733-890F-1A7896418D71

Tylopus brehieri Golovatch, VandenSpiegel & Semenyuk, 2016: 335 (D).

###### Record from Myanmar.

Shan State, Kyauk Khaung (= Stone Cave) (Golovatch et al. 2016).

##### Tylopus doriae

Taxon classificationAnimaliaPolydesmidaParadoxosomatidae

(Pocock, 1895)

176798B6-F3E2-5413-A8CE-069CD6BC77DC

Orthomorpha doriae Pocock, 1895: 823 (D).
Orthomorpha Doriae (sic!) – Attems, 1898: 339 (L, K).
Orthomorpha (Kalorthomorpha) doriae – Attems, 1936: 204 (L).
Orthomorpha (Orthomorpha) doriae – Attems, 1937: 80 (D, K).
Orthomorpha doriae – Weidner, 1960: 85 (L).
Agnesia doriae – Jeekel, 1965: 100 (D, K).
Tylopus doriae – Jeekel, 1968: 60 (M); Golovatch and Enghoff 1993: 103 (D, K); Enghoff 2005: 99 (R); Likhitrakarn et al. 2010: 25 (L, K); 2014: 65 (L, K); 2016: 35 (L, K); Nguyen and Sierwald 2013: 1298 (L).

###### Records from Myanmar.

Yado, 1000–1400 m; Bia-Po, 1000–1200 m, Meteleo, 900–1200; Puepoli, 900–1200 m (Pocock 1895).

###### Remark.

Also found in Doi Suthep National Park (1400–1500 m), Chiang Mai Province, Thailand (Enghoff 2005).

##### Tylopus punctus

Taxon classificationAnimaliaPolydesmidaParadoxosomatidae

Likhitrakarn, Golovatch & Panha, 2016

F1039630-1812-5313-ABB0-20FFEF1C86AB

Tylopus punctus Likhitrakarn, Golovatch & Panha, 2016: 29 (D).

###### Record from Myanmar.

Mintaingbin Forest Camp, ca 35 km north of Aungban, Chan State, 20°55'20"N, 96°33'60"E, ca 1300 m a.s.l. (Likhitrakarn et al. 2016).

##### Tylopus silvestris

Taxon classificationAnimaliaPolydesmidaParadoxosomatidae

(Pocock, 1895)

868F6F93-C0FC-5BD3-9F8C-165C532915FE

Orthomorpha silvestris Pocock, 1895: 824 (D).
Orthomorpha silvestris – Attems, 1914: 238 (L); 1936: 205 (L); 1937: 94 (L).
Agnesia silvestris – Jeekel, 1965: 104 (D, K).
Tylopus silvestris – Jeekel 1968: 60 (M); Golovatch and Enghoff 1993: 90 (M, K); Likhitrakarn et al. 2010: 26 (L, K); 2016: 38 (L, K); Nguyen and Sierwald 2013: 1300 (L).

###### Record from Myanmar.

Village of Thao (Carin Ghecu, 1200–1400 m) (Pocock 1895).

##### Tylopus monticola sp. nov.

Taxon classificationAnimaliaPolydesmidaParadoxosomatidae

21DBC0B8-DDB2-50D3-AC18-055CCDC0A131

http://zoobank.org/FD497FBD-67B7-4171-9905-ED6D2011DF51

Figs 1A , 2 , 3 , 4

###### Material examined.

***Holotype*:** Myanmar – **Shan State** • ♂; Taunggyi District, near Montawa Cave; elev. 1204 m; 20°45'15.9"N, 97°01'03.4"E; 21 Sep. 2016; J. Sutcharit, R. Srisonchai leg.; CUMZ. ***Paratypes***: Myanmar – **Shan State** • 3 ♀♀; same collection data as holotype; CUMZ • 1 ♀; same collection data as holotype; ZMUM • 3 ♀♀; near Aye Say Tee Cave; elev. 1583 m; 20°47'29.5"N, 97°03'01.6"E; 21 Sep. 2016; J. Sutcharit, R. Srisonchai leg.; CUMZ • 1 ♂; Parpant area, outside the cave; elev. 1159 m; 20°15'03.7"N, 97°14'23.9"E; 23 Sep. 2016; J. Sutcharit, R. Srisonchai leg.; CUMZ • 1 ♂; same collection data as previous; ZMUM.

###### Diagnosis.

Using the latest key to *Tylopus* species (Likhitrakarn et al. 2016), as well as the information concerning all 12 congeners described since (Golovatch et al. 2016; Golovatch 2018, 2019, 2020; Golovatch and Semenyuk 2018), *T. monticola* sp. nov. keys out to *T. rugosus* Golovatch & Enghoff, 1993 on account of the particularly strong similarities in the gonopodal structure (Fig. 8). Thus, even though they both share most of the somatic and gonopodal features, the new species differs in the large and long process **z** with a serrate edge along the dorsal margin, which protrudes beyond the apicolateral lobe (**l**) (Figs 3B–D, 4A, B) (vs. smaller and not protruding beyond **l**) (Fig. 8B, C), all ♂ legs with the prefemora swollen laterally except for leg 1 (vs. except for legs 1 and 2), coupled with the pleurosternal carinae complete crests with an evident, sharp, caudal denticle produced past the rear tergal margin on segments 4–7, gradually decreasing in size until segments 15(16) (♂) or 13(12) (♀) (Fig. 2B, D, E) (vs. same, but gradually decreasing in size until segment 18).

###### Description.

Length of holotype ca 30 mm; adult paratypes 29–31 (♂) or 32–35 mm (♀), width of midbody pro- and metazonae of holotype, 2.4 and 3.6 mm; adult paratypes 2.4–2.5 and 3.4–3.7 mm (♂) or 2.9–3.5 and 3.7–4.5 mm (♀), respectively.

**Figure 1.**
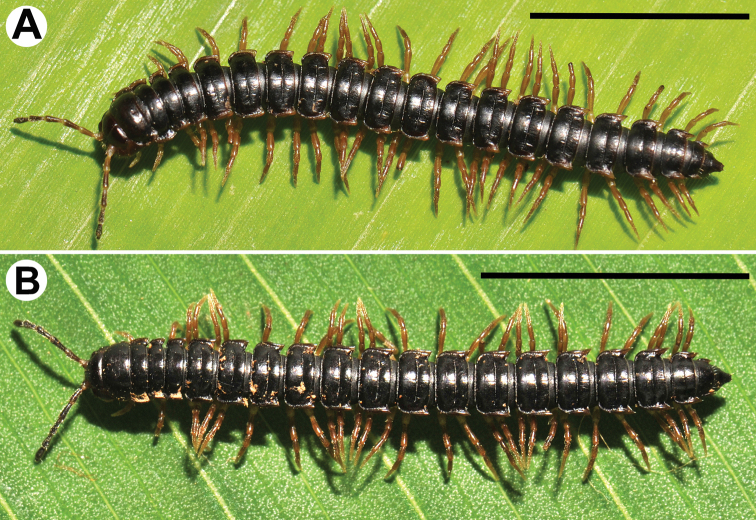
Habitus, live colouration **A***Tylopus monticola* sp. nov., ♂ paratype (CUMZ) **B***Tylopus sutchariti* sp. nov., ♂ holotype (CUMZ). Scale bars: 1 cm.

**Figure 2.**
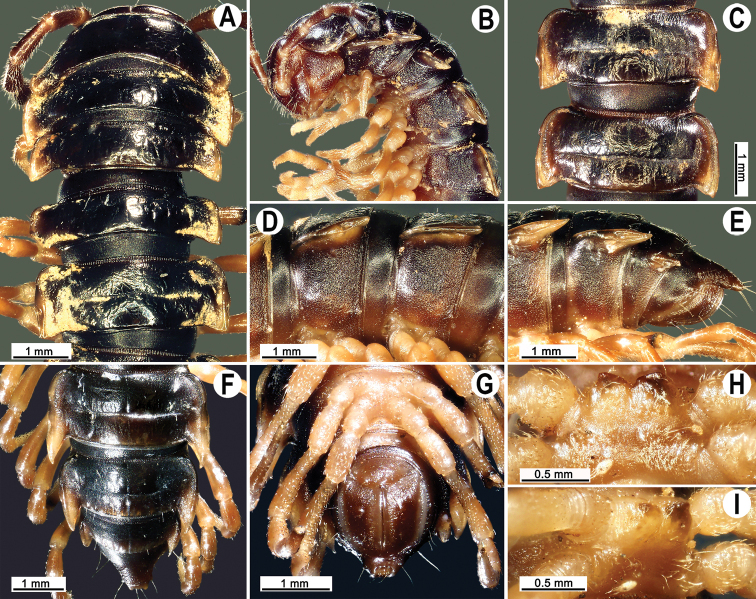
*Tylopus monticola* sp. nov., ♂ holotype (CUMZ) **A, B** anterior part of body, dorsal and lateral views, respectively **C, D** segments 10 and 11, dorsal and lateral views, respectively **E–G** posterior part of body, lateral, subdorsal and subventral views, respectively **H, I** sternal cones between coxae 4, subcaudal and sublateral views, respectively.

Colouration of live animals dark brown (Fig. 1A); calluses of paraterga, venter and legs lighter brown; colouration of alcohol material after two years of preservation faded to dark brown; head, antennae and tip of epiproct light brown, calluses of paraterga yellowish brown to pallid, venter and legs light brown to light yellowish (Fig. 2).

Clypeolabral region and vertex sparsely setose, epicranial suture distinct. Antennae short (Figs 1A, 2A, B), reaching body segment 3 (♂) or 2 (♀) when stretched dorsally. In width, head < segment 3 < 4 < 5 < 6 < collum < segment 2 < 7–16 (♂, ♀); thereafter body gently and gradually tapering. Collum with three transverse rows of strong setae: 3+3 anterior, 2+2 intermediate, and 3+3 posterior; a small lateral incision near midway; caudal corner of paraterga rounded, slightly declined ventrad, not produced past rear tergal margin (Fig. 2A, B).

Tegument rather smooth and shining, prozonae very finely shagreened, metaterga mainly smooth, but often rugulose; surface below paraterga finely microgranulate (Fig. 2A–F). Postcollum metaterga with two transverse rows of rather long setae: 2+2 in anterior and 3+3 in posterior row, the latter often abraded, but then readily traceable as insertion points. Tergal setae long, strong, slender, about 1/3 metatergal length. Axial line visible both on pro- and metazonae.

Paraterga strongly developed (Fig. 2A–F), especially well so in ♂, subhorizontal, slightly upturned posteriorly, always lying high, at upper 1/3 of midbody height, but remaining below dorsum; anterior edge well-developed, mostly regularly rounded and narrowly bordered, fused to callus; caudal corner narrowly rounded, extending increasingly past rear tergal margin, especially strongly so on segments 15–19; in segments 16–19, tips strongly curved mesad, posterior edge slightly oblique (Fig. 2A, C, F); paraterga very thin blunt blades in lateral view, a little thicker only on pore-bearing segments (Fig. 2D). Calluses on paraterga delimited by a sulcus only dorsally. Paraterga 2 broad, lateral edge with three evident incisions: one in anterior 1/3, one at midway, and one at posterior 1/3; anterior incision particularly evident. Paraterga 3 and 4 with two small incisions at lateral edge (Fig. 2A), one in anterior 1/3, the other at posterior 1/3; anterior incision also particularly evident. Lateral edge of paraterga of following segments with two small incisions, one in anterior 1/3, the other at midway, caudal incision being smaller in pore-bearing segments (Fig. 2C). Ozopores evident, lateral, lying in an ovoid groove at about 1/3 metatergal length in front of posterior edge of metaterga (Fig. 2D). Transverse sulcus usually distinct (Fig. 2A, C, F), slightly incomplete on segment 18, complete and clearly visible on metaterga 5–17, deep, reaching the bases of paraterga, arcuate, faintly beaded at bottom. Stricture between pro- and metazonae narrow, shallow, beaded at bottom down to base of paraterga (Fig. 2A, C, F). Pleurosternal carinae complete crests on segment 2–3(4) (Fig. 2B), with an evident and sharp denticle caudally on segments 4(5)–7 (♂, ♀), thereafter increasingly well reduced and remaining only a small sharp caudal tooth until segment 15(16) (♂) or 13(12) (♀), thereafter missing (Fig. 2B, D, E). Epiproct (Fig. 2E–G) conical, flattened dorsoventrally, subtruncate, with two evident apical papillae directed caudally, both pointed at tip; pre-apical papillae evident, lying close to tip. Hypoproct subtrapeziform (Fig. 2G), small setiferous knobs at caudal edge well-separated and evident.

Sterna densely setose, without modifications (Fig. 2G); cross-impressions shallow; a deeply notched sternal lobe between ♂ coxae 4 (Fig. 2H, I). Legs long and slender, midbody ones ca 1.4–1.5 (♂) or 0.9–1.0 (♀) as long as body height; all ♂ legs except leg 1 with prefemora swollen laterally; femora and tibiae with particularly dense setae and ventral microgranulations; legs on segments 7–18 with an evident adenostyle (tubercle) medially on each postfemur and tibia (Fig. 4C); tarsal brushes absent.

**Figure 3.**
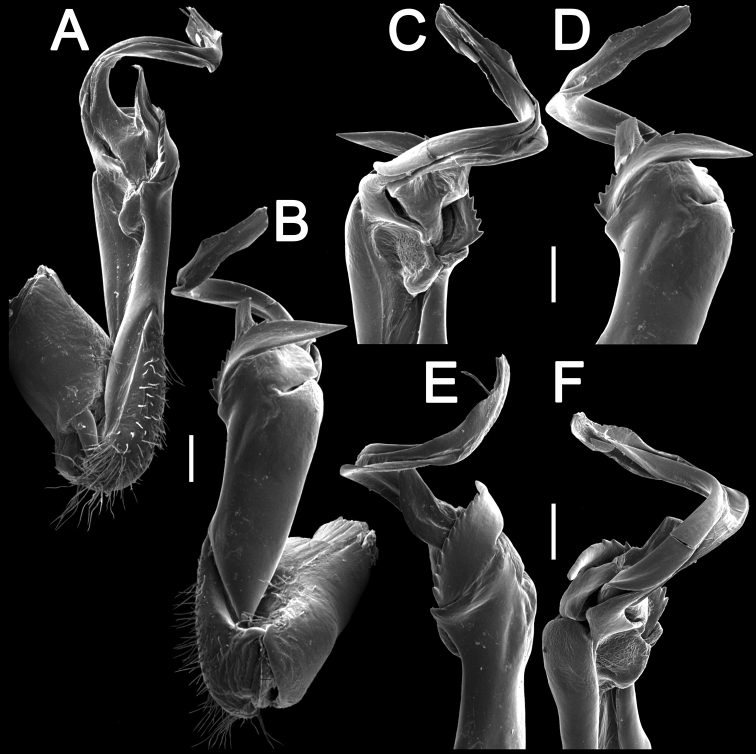
*Tylopus monticola* sp. nov., ♂ holotype (CUMZ), left gonopod **A, B** mesal and lateral views, respectively **C–F** distal part, submesal, lateral, suboral and subcaudal views, respectively. Scale bars: 0.2 mm.

**Figure 4.**
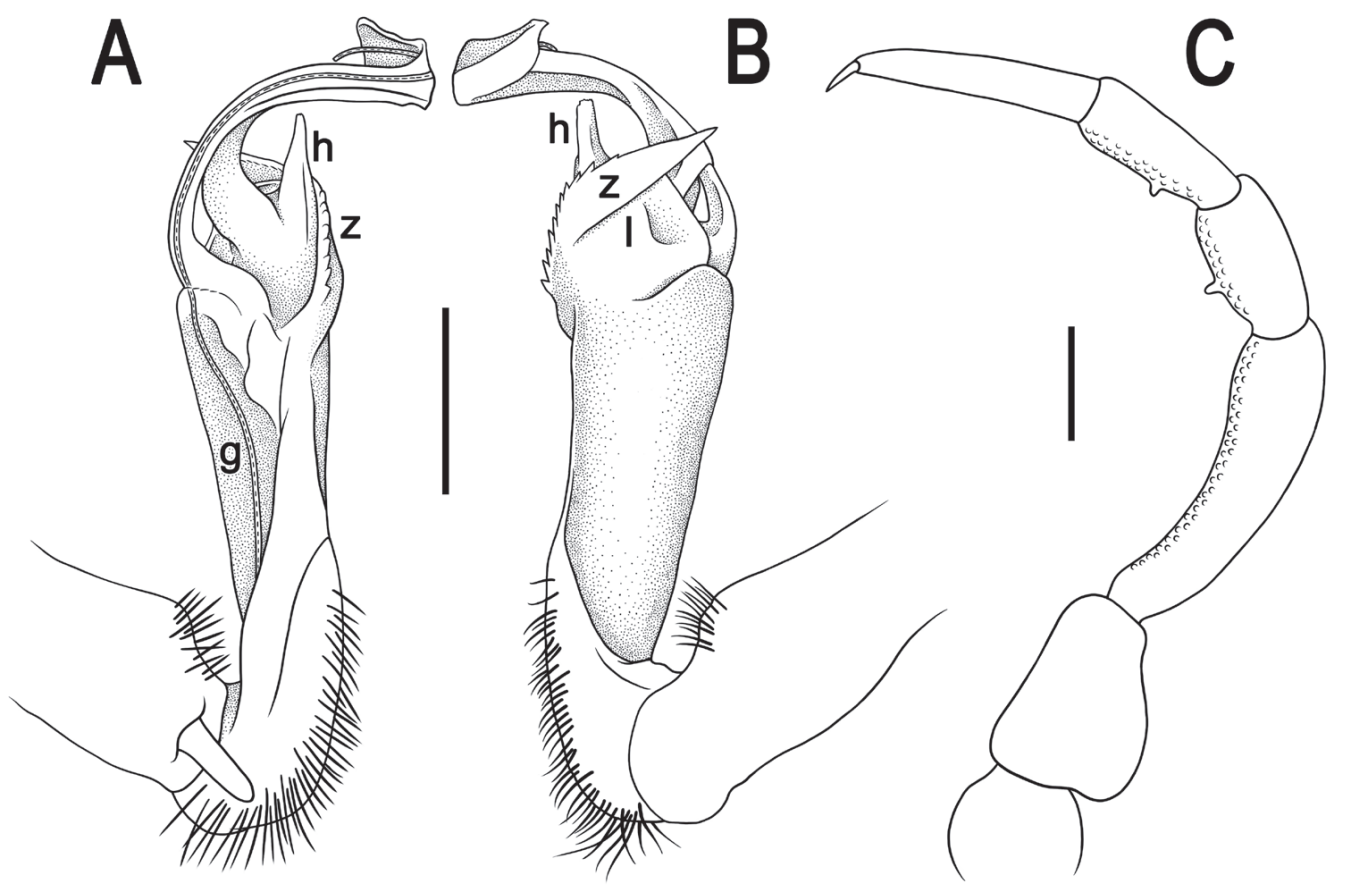
*Tylopus monticola* sp. nov., ♂ holotype (CUMZ) **A, B** left gonopod, mesal and lateral views, respectively **C** leg of segment 10. Abbreviations: **g** mesal groove/hollow of femorite, **h** short and stout process of femorite, **l** apicolateral lobe of femorite, **z** serrate process of femorite. Scale bars: 0.5 mm.

Gonopods (Figs 3, 4A, B) simple; coxite slightly curved caudad, sparsely setose distoventrally. Prefemorite densely setose, about 1/3 as long as femorite + “postfemoral” part. Femorite rather stout, expanded distad, suberect, showing a distinct mesal groove/hollow (**g**); apicolateral lobe (**l**) simple; process **z** large and long, serrate along dorsal margin and protruding beyond apicolateral lobe (**l**); process **h** short and stout, suberect, with a narrowly rounded tip; solenophore long and slender, typically coiled, tip subtruncate.

###### Name.

To emphasize the habitats where this new species was discovered; “*monticola*” meaning a mountain-dweller or a highlander; noun in apposition.

###### Remark.

The species was found quite far away (about 120 air-km) from the type locality of the most similar species, *T. rugosus* Golovatch & Enghoff, 1993 (Fig. 9). Both new species described here have been found to occur syntopically.

##### Tylopus sutchariti sp. nov.

Taxon classificationAnimaliaPolydesmidaParadoxosomatidae

B4DFAF16-7488-5A80-AFDE-56D716EED2C4

http://zoobank.org/5385A3A0-C129-41F3-9A8C-C3910EF9178C

Figs 1B , 5 , 6 , 7

###### Material examined.

***Holotype*:** Myanmar – **Shan State** • ♂; Taunggyi District, near Montawa Cave; elev. 1204 m; 20°45'15.9"N, 97°01'03.4"E; 21 Sep. 2016; R. Srisonchai leg.; CUMZ. ***Paratype***: Myanmar – **Shan State** • 1 ♀; same collection data as holotype; CUMZ.

###### Diagnosis.

**Figure 5.**
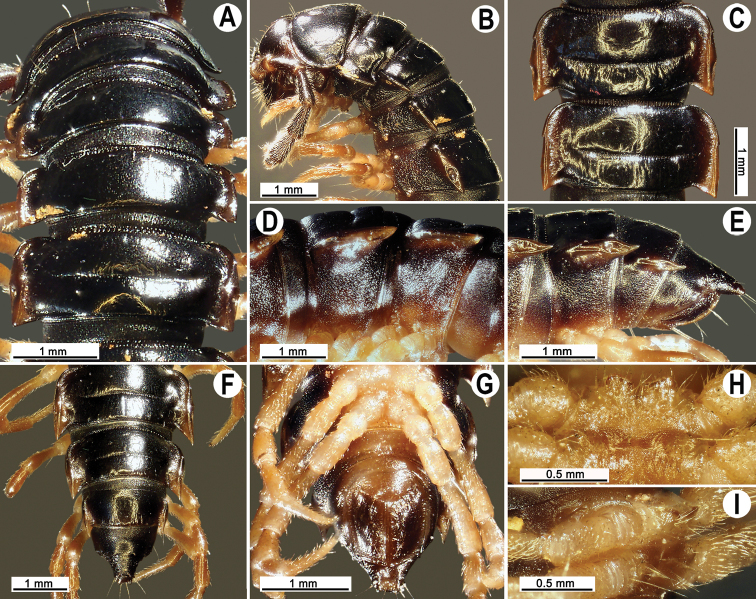
*Tylopus sutchariti* sp. nov., ♂ holotype (CUMZ) **A, B** anterior part of body, dorsal and lateral views, respectively **C, D** segments 10 and 11, dorsal and lateral views, respectively **E–G** posterior part of body, lateral, subdorsal and subventral views, respectively **H, I** sternal cones between coxae 4, subcaudal and sublateral views, respectively.

**Figure 6.**
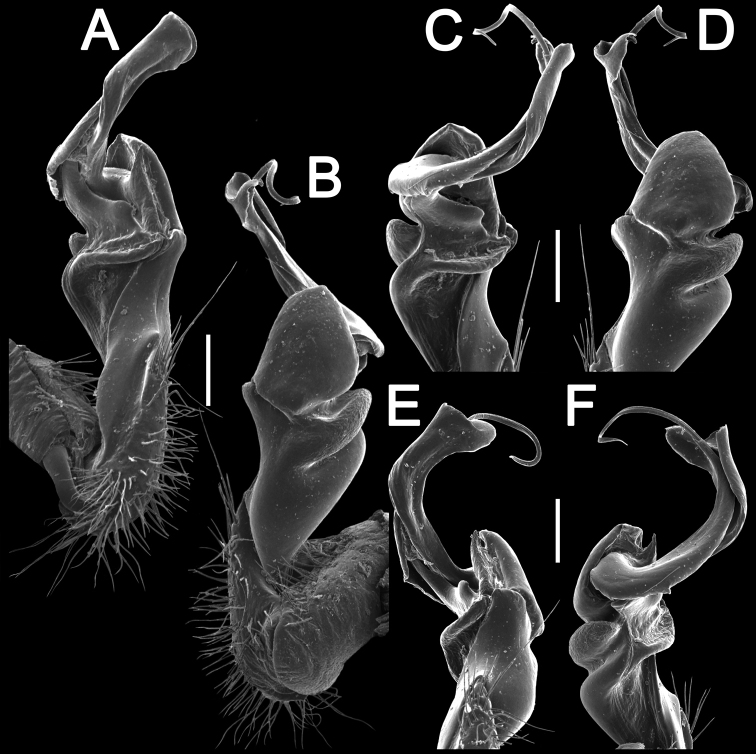
*Tylopus sutchariti* sp. nov., ♂ holotype (CUMZ), left gonopod **A, B** mesal and lateral views, respectively **C–F** distal part, submesal, sublateral, suboral and subcaudal views, respectively. Scale bars: 0.2 mm.

**Figure 7.**
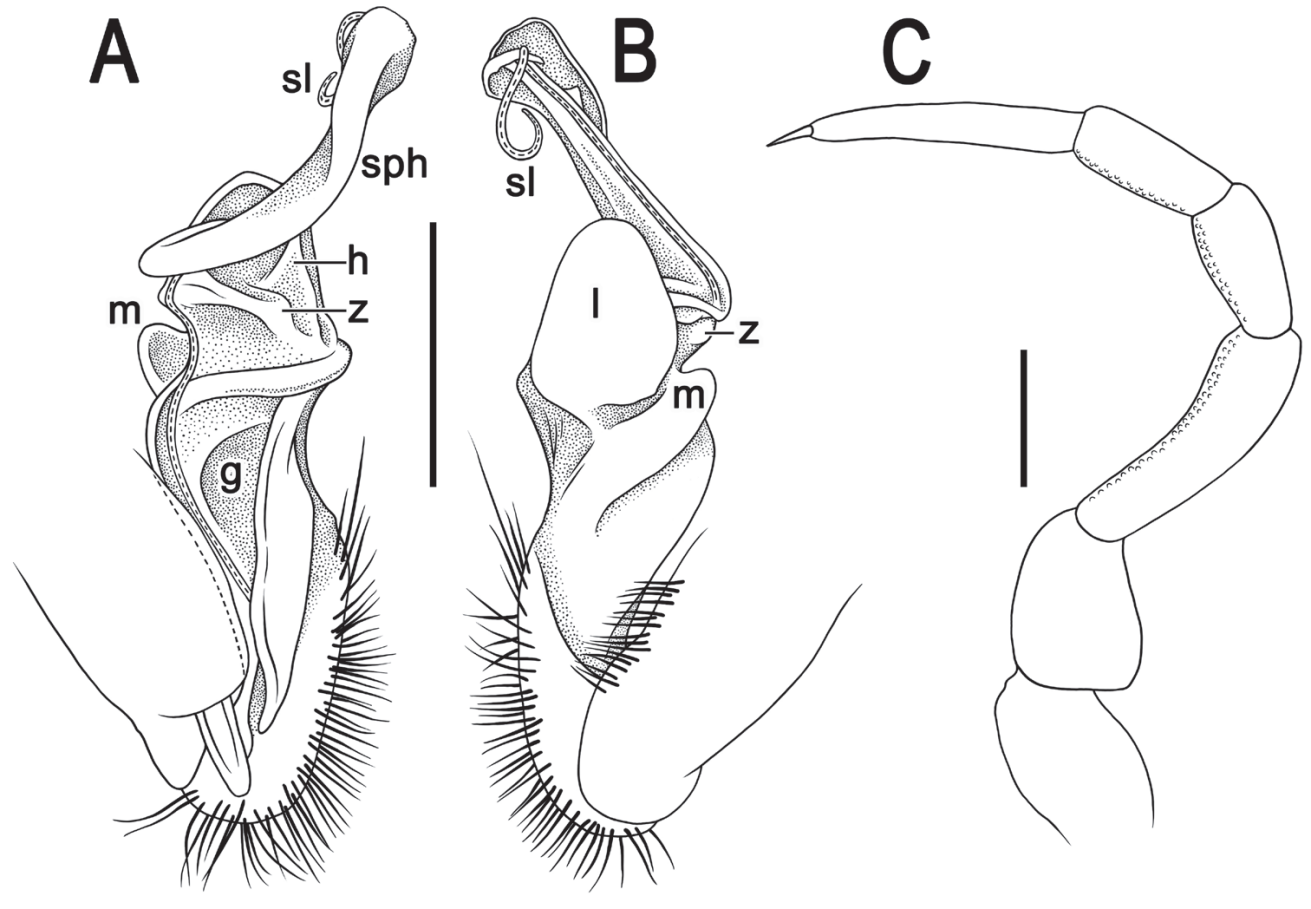
*Tylopus sutchariti* sp. nov., ♂ holotype (CUMZ) **A, B** left gonopod, mesal and lateral views, respectively **C** leg of segment 10. Abbreviations: **g** mesal groove/hollow of femorite, **h** short and triangular process of femorite, **l** apicolateral lobe of femorite, **m** dorsoapical lobe of femorite, **sl** tip of solenomere, **sph** solenophore, **z** short and simple process of femorite. Scale bars: 0.5 mm.

This new species comes to a dead end in couplet 5 in the latest key to *Tylopus* species (Likhitrakarn et al. 2016), but it seems to be particularly similar to the later described and grossly sympatric *T. brehieri* Golovatch, VandenSpiegel & Semenyuk, 2016, especially in its gonopod conformation. Both species compared come from Shan State, Myanmar (Golovatch et al. 2016), but *T. sutchariti* sp. nov. differs in the presence of a small and triangular gonopod process **h** (vs. absent), and the large and subtrapeziform apicolateral lobe (**l**) with a smooth apical margin (Figs 6B, D, 7B) (vs. a subtriangular **l** with an apically rugose and denticulate margin), as well as the pleurosternal carinae being complete crests with a caudal tooth clearly visible until segments 16 (♂) or 13 (♀), thereafter missing (Fig. 5B, D, E) (vs. visible until segment 10), while the sternal lobe between ♂ coxae 4 is deeply notched (Fig. 5H, I) (vs. prominent and subquadrate).

###### Description.

Length 24.1 (♂) or 22.5 mm (♀), width of midbody pro- and metazona 1.8 and 2.7 mm (♂) or 2.2 and 2.6 mm (♀), respectively.

Colouration of live animals dark brown (Fig. 1B); venter and legs brown; colouration of alcohol material after two years of preservation blackish, calluses of paraterga yellowish brown, head and antennae dark brown, venter and legs light yellowish, increasingly darker brown distally (Fig. 5).

Clypeolabral region and vertex sparsely setose, epicranial suture distinct. Antennae short (Figs 1B, 5B), reaching body segment 3 (♂) or surpassing body segment 2 (♀) when stretched dorsally. In width, head < segment 3 < 4 < 5 < collum < segment 2 < 6–17 (♂, ♀); thereafter body gently and gradually tapering. Collum with three transverse rows of strong setae: 3+3 anterior, 1+1 intermediate, and 3+3 posterior; a small lateral incision near midway; caudal corner of paraterga rounded, slightly declined ventrad, not surpassing rear tergal margin (Fig. 5B).

Tegument rather smooth and shining, prozonae very finely shagreened, metaterga smooth and finely rugulose; surface below paraterga finely microgranulate (Fig. 5A–F). Postcollum metaterga with two transverse rows of rather long setae: 2+2 in anterior and 2(3)+2(3) in posterior row, the latter often abraded, but then readily traceable as insertion points. Tergal setae long, strong, slender, about 1/3 metatergal length. Axial line visible only on metazonae.

Paraterga strongly developed (Fig. 5A–F), especially well so in ♂, set high, at upper 1/3 of midbody height, slightly upturned, but remaining below dorsum; anterior edge well-developed, mostly regularly rounded and narrowly bordered, continuous with callus; caudal corner narrowly rounded to fully pointed, extending increasingly past rear tergal margin, especially well so on segments 15–19; on segments 16–19, tips strongly curved mesad, posterior edge slightly oblique (Fig. 5A, C, F); paraterga very thin blunt blades in lateral view, a little thicker only on pore-bearing segments (Fig. 5D). Calluses on paraterga delimited by a sulcus both dorsally and ventrally. Paraterga 2 broad, horizontal, anterior edge angular, lateral edge with three evident incisions, one in anterior 1/3, middle one at midway, caudal incision near tip; anterior incision particularly evident. Paraterga 3 and 4 with two small incisions at lateral edge (Fig. 5A), one in anterior 1/3, the other at midway, anterior one also particularly evident. Following segments each with lateral edge showing an evident incision near front 1/3 (Fig. 5C). Ozopores evident, lateral, lying in an ovoid groove at about 1/3 metatergal length in front of posterior edge of metaterga (Fig. 5D). Transverse sulcus usually distinct (Fig. 5A, C, F), complete and visible on metaterga 5–18, deep, narrow, reaching bases of paraterga, line-shaped, clearly beaded at bottom. Stricture between pro- and metazonae wide, deep, clearly ribbed at bottom down to base of paraterga (Fig. 5A, C, F). Pleurosternal carinae complete crests on segment 2–4 (Fig. 5B), with anteriorly bulged crests and a sharp denticle caudally on segments 5–8 (♂, ♀), thereafter increasingly reduced and broken, remaining only a small sharp caudal tooth until segment 16 (♂) or 13 (♀), thereafter missing (Fig. 5B, D, E). Epiproct (Fig. 5E–G) conical, flattened dorsoventrally, subtruncate, with two evident apical papillae directed caudally, both pointed at tip; pre-apical papillae evident, lying close to tip. Hypoproct roundly subtrapeziform (Fig. 5G), small setiferous knobs at caudal edge well-separated and evident.

Sterna densely setose, without modifications (Fig. 5G); cross-impressions shallow; a deeply notched sternal lobe between ♂ coxae 4 (Fig. 5H, I). Legs long and slender, midbody ones ca 1.4–1.5 (♂) or 1.1–1.2 (♀) as long as body height; ♂ legs of segments 4–17 with prefemora distinctly swollen laterally; ♂ legs of segments 2–16 each with femur, postfemur, tibia and tarsus with particularly dense setae and carrying ventral microgranulations (Fig. 7C), tarsal brushes absent.

Gonopods (Figs 6, 7A, B) complex; coxa slightly curved caudad, sparsely setose distoventrally. Prefemorite as usual, densely setose, about 1/2 as long as femorite + “postfemoral” part. Femorite rather stout, suberect, expanded distad, showing a distinct mesal groove/hollow (**g**) and a prominent, rounded, dorso-apical lobe (**m**), apicolateral lobe (**l**) large, subtrapeziform, with a smooth apical margin, mostly delimited at base by a transverse sulcus, with process **h** short and triangular; process (**z**) rather short and simple, narrowly rounded at tip. Solenophore (**sph**) typically coiled, lamellar, expanded apically into a subtruncate lobe, almost fully sheathing a similarly long, flagelliform solenomere, with only its tip (**sl**) being exposed.

###### Name.

To honour Dr. Chirasak Sutcharit, Professor at the Department of Biology of the Chulalongkorn University, Bangkok, who participated in collecting the type series.

**Figure 8.**
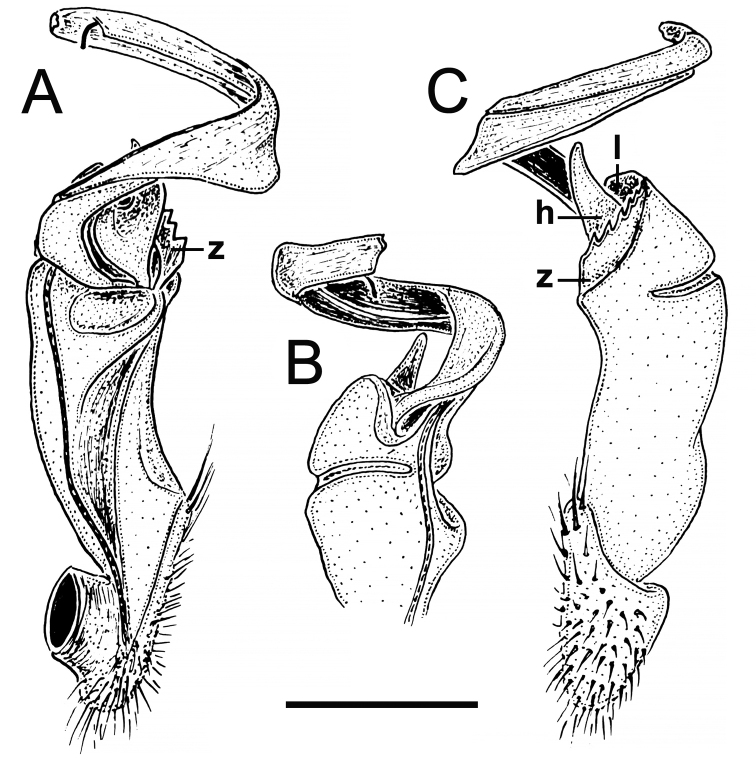
Gonopod structure of *Tylopus rugosus* Golovatch & Enghoff, 1993, ♂ holotype, left gonopod **A–C** mesal, lateral and dorsal views, respectively. Abbreviations: **h** strong hook-shaped process of femorite, **l** apicolateral lobe of femorite, **z** serrate process of femorite. Scale bar: 0.5 mm (after Golovatch and Enghoff 1993).

###### Remark.

**Figure 9.**
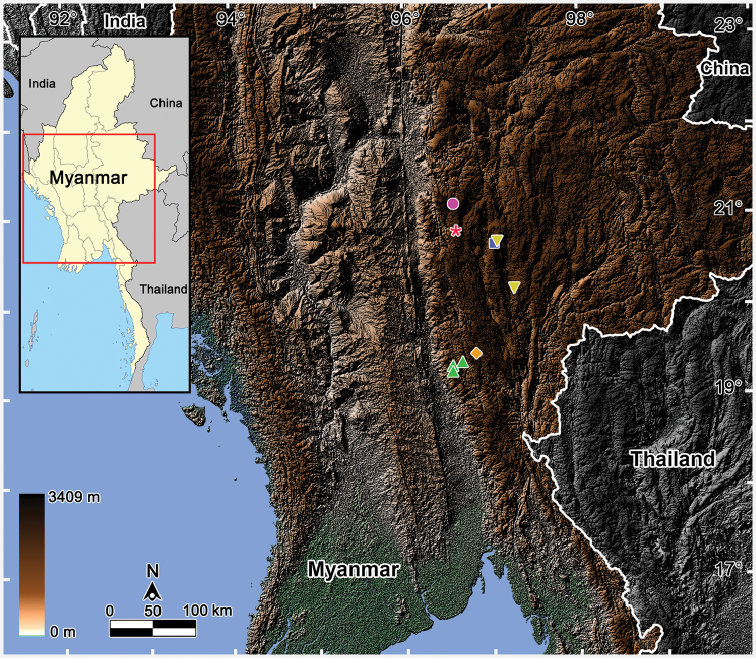
Distributions of *Tylopus* species recorded from Myanmar. Pink circle: *T. brehieri* Golovatch, VandenSpiegel & Semenyuk, 2016; Red Asterisk: *T. punctus* Likhitrakarn, Golovatch & Panha, 2016; Yellow inverted triangle: *T. monticola* sp. nov.; Blue square: *T. sutchariti* sp. nov. and *T. monticola* sp. nov.; Orange diamond: *T. silvestris* (Pocock, 1895); Green triangle: *T. doriae* (Pocock, 1895).

Both new species described here have been found to occur syntopically (Fig. 9).

##### Key to species of *Tylopus* currently known to occur in Myanmar, chiefly based on ♂ characters

**Table:** 

1	All ♂ prefemora normal, not bulged laterally	**2**
–	Most ♂ prefemora clearly swollen laterally (Figs 4C, 7C)	**3**
2	Body smaller: width up to 2.1–2.5 mm. Midbody paratergal corner very narrowly rounded and not protruding caudad past rear margin. Gonopod process **h** small and pointed	***Tylopus punctus* Likhitrakarn, Golovatch & Panha, 2016**
–	Body larger: width 3.0 mm. Midbody paratergal corner nearly pointed and protruding caudad past rear margin. Gonopod process **h** absent	***T. silvestris* (Pocock, 1895)**
3	Paratergal calluses with only one incision. Gonopod postfemoral lobe **l** much longer than broad; area basal to **l** delimited by a distinct cingulum (Figs 6B, D, 7B)	**4**
–	Paratergal calluses with two incisions. Gonopod postfemoral lobe **l** either as long as broad or longer; no cingulum basal to **l** (Figs 3B, D, 4B)	**5**
4	Sternal lobe between ♂ coxae 4 deeply notched (Fig. 5H, I). Gonopod process **h** small and triangular, while apicolateral lobe (**l**) large and subtrapeziform with a smooth apical margin (Figs 6B, D, 7B)	***T. sutchariti* sp. nov.**
– Sternal lobe between ♂ coxae 4 prominent and subquadrate. Gonopod process **h** absent, while apicolateral lobe (**l**) subtriangular with an apically rugose and denticulate margin	***T. brehieri* Golovatch, VandenSpiegel & Semenyuk, 2016**	
5	Gonopod process **z** large and long, protruding beyond apicolateral lobe (**l**) (Figs 3B–D, 4B). All ♂ legs with prefemora swollen laterally except for leg 1. Pleurosternal carinae present before segment 16	***T. monticola* sp. nov.**
–	Gonopod process **z** smaller and not protruding beyond apicolateral lobe (**l**) (Fig. 8B, C). All ♂ legs with prefemora swollen laterally except for legs 1 and 2. Pleurosternal carinae present until segment 18	***T. rugosus* Golovatch & Enghoff, 1993**

## Discussion

Of a total of 76 species of *Tylopus* presently known globally, including two new described above, most of the diversity (31 species, or >41%) comes from Thailand, followed by Vietnam (21 species), Laos (12 species), southern China (8 species) and Myanmar (6 species). Almost all *Tylopus* species appear to be confined to montane woodlands exceeding 500 m in elevation (Likhitrakarn et al. 2016). Furthermore, most of them (92%) are short-range endemics or confined to a small area (< 4000 km^2^). Many species occur sympatrically, some even syntopically, but then they tend to differ in the timing of sexual maturity or mating season. For instance, the Doi Inthanon and Doi Suthep mountains, both in northern Thailand, support at least 10 congeners each (Likhitrakarn et al. 2014). Unfortunately, most of the known species (75%) have only been collected once and from a single locality.

The genus *Tylopus* seems to be particularly similar to two genera of the large and mostly Asian tribe Sulciferini, viz. *Oxidus* Cook, 1911 and *Hedinomorpha* Verhoeff, 1934. All three share the presence of a unique gonopodal apicolateral lobe (**l**) separated from the femorite by a more or less distinct, basal, (sub)transverse sulcus. Golovatch (2021) has recently discussed the morphological differences between these three genera, *Tylopus* being distinct primarily in the particularly elaborate gonopodal telopodite. The distinction of *Tylopus* from *Oxidus* has also been confirmed by molecular evidence (Nguyen et al. 2017).

In addition to putting on record two new, presumably narrowly endemic species of *Tylopus* from the Taunggyi Mountains, southwestern Shan State, Myanmar, and thus bringing the number of *Tylopus* spp. of Myanmar to a total of six, we map their distributions (Fig. 9). Only *T. doriae* has been recorded from two countries, Myanmar and Thailand, whereas the other five seem to be more strongly localized. Given that Myanmar remains one of the largest, but least-surveyed countries in the East Indies, and considering the large limestone montane areas it harbours, there can hardly be any doubt that more species of *Tylopus* will be found there in the future.

## Supplementary Material

XML Treatment for Tylopus brehieri

XML Treatment for Tylopus doriae

XML Treatment for Tylopus punctus

XML Treatment for Tylopus silvestris

XML Treatment for Tylopus monticola

XML Treatment for Tylopus sutchariti
